# Enhanced Diaphragm Muscle Function upon Satellite Cell Transplantation in Dystrophic Mice

**DOI:** 10.3390/ijms25052503

**Published:** 2024-02-21

**Authors:** Karim Azzag, Heather M. Gransee, Alessandro Magli, Aline M. S. Yamashita, Sudheer Tungtur, Aaron Ahlquist, Wen-Zhi Zhan, Chiemelie Onyebu, Sarah M. Greising, Carlos B. Mantilla, Rita C. R. Perlingeiro

**Affiliations:** 1 Lillehei Heart Institute, Department of Medicine, University of Minnesota, Minneapolis, MN 55455, USA; kazzag@umn.edu (K.A.); alemagli@gmail.com (A.M.); sakag012@umn.edu (A.M.S.Y.); stungtur@gmail.com (S.T.); ahlqu043@umn.edu (A.A.); onyeb002@umn.edu (C.O.); 2 Department of Anesthesiology and Perioperative Medicine, Mayo Clinic, Rochester, MN 55905, USA; gransee.heather2@mayo.edu (H.M.G.); zhan.wenzhi@mayo.edu (W.-Z.Z.); mantilla.carlos@mayo.edu (C.B.M.); 3 School of Kinesiology, University of Minnesota, Minneapolis, MN 55455, USA; grei0064@umn.edu; 4 Department of Physiology and Biomedical Engineering, Mayo Clinic, Rochester, MN 55905, USA; 5 Stem Cell Institute, University of Minnesota, Minneapolis, MN 55455, USA

**Keywords:** diaphragm, muscular dystrophy, transplantation, muscle stem cells, satellite cells, pre-injury, engraftment, regeneration, specific force

## Abstract

The diaphragm muscle is essential for breathing, and its dysfunctions can be fatal. Many disorders affect the diaphragm, including muscular dystrophies. Despite the clinical relevance of targeting the diaphragm, there have been few studies evaluating diaphragm function following a given experimental treatment, with most of these involving anti-inflammatory drugs or gene therapy. Cell-based therapeutic approaches have shown success promoting muscle regeneration in several mouse models of muscular dystrophy, but these have focused mainly on limb muscles. Here we show that transplantation of as few as 5000 satellite cells directly into the diaphragm results in consistent and robust myofiber engraftment in dystrophin- and fukutin-related protein-mutant dystrophic mice. Transplanted cells also seed the stem cell reservoir, as shown by the presence of donor-derived satellite cells. Force measurements showed enhanced diaphragm strength in engrafted muscles. These findings demonstrate the feasibility of cell transplantation to target the diseased diaphragm and improve its contractility.

## 1. Introduction

A significant number of disorders affect diaphragm muscle function, leading to reduced quality of life and often early death [1]. Among these are many types of muscular dystrophy (MD), a heterogeneous group of genetic disorders characterized by progressive muscle wasting, in which diaphragm and/or heart involvement is associated with cardio-respiratory failure [2]. While the diaphragm muscle is spared or rarely affected in some types of MD, such as limb-girdle MD type 2A (LGMD2A, also known as LGMDR1) [3] and facioscapulohumeral MD (FSHD) [4], this is not the case for many types of MD. For instance, respiratory complications are a prominent disease phenotype of the two most prevalent types of MD, myotonic dystrophy type I (DM1) and Duchenne MD (DMD). The percentages of death due to respiratory complications have been reported to be 43% for DM1 [5] and 38% for DMD [6]. Mechanical ventilation has helped DMD patients to live into adulthood, as prior to this, most patients succumbed by their teenage years due to respiratory failure [7]. Mutations in fukutin-related protein (FKRP), which encompass a heterogeneous spectrum of dystroglycanopathies, ranging from limb-girdle MD type 2I (LGMD2I, also known as LGMDR9) to several forms of congenital MD (MDC1C) [8,9,10], are also characterized by respiratory impairment due to loss of diaphragm function. A study compiling 36 LGMD2I patients reported that about half displayed respiratory dysfunction at an average age of 32 years [11].

Although not abundant, there have been studies assessing therapeutic approaches to circumvent diaphragm dysfunction in relevant dystrophic mouse models. Among these are studies of anti-inflammatory [12,13] and anti-oxidant [14,15] drugs in the mdx mouse model of DMD. In the case of LGMD2I, two studies have shown improvement in the respiratory function of FKRP-mutant mice (FKRP^P448L^), one in response to ribitol treatment [16] and the other to a combination of tamoxifen and raloxifene [17]. These treatments may improve the patient’s quality of life, but they are not curative, and thus, other therapeutic approaches are being pursued.

Currently, most translational efforts are focused on gene therapy, and some preclinical studies for DMD and LGMD2I include diaphragm muscle assessment. For DMD, an early study showed improvement of respiratory function upon injection of mdx/utrophin^−/−^ mice with a full-length dystrophin expression cassette [18]. Recent studies involving *adeno*-*associated virus* (*AAV*)-mediated *micro-dystrophin* gene therapy in mdx mice [19] and a DMD dog model [20] demonstrated rescue of diaphragm dystrophin expression and amelioration of the respiratory phenotype. Regarding LGMD2I, the systemic delivery of *AAV*-mediated human FKRP to the FKRP^P448L^ mouse model also resulted in improvement of respiratory function [21]. Although gene therapy holds great promise to treat MDs, there remain some challenges to be circumvented, such as the control of the immune response, as indicated by recent clinical trials [22].

Cell therapy is also considered an attractive therapeutic option for MDs. Several cell types, including muscle stem cells, mesoangioblasts, and pluripotent stem cell-derived myogenic progenitors, among others, have been documented to contribute to muscle regeneration and rescue of the dystrophic phenotype, in many cases with long-term potential [23]. Most studies have made use of intramuscular transplantation [24,25,26,27,28,29,30,31,32], but systemic delivery has also been reported [26,33,34,35,36,37,38]. To date, studies have targeted almost exclusively the hind limb muscles (the tibialis anterior muscle is a favorite due to its accessibility), with only a few reports describing cell engraftment in the main respiratory muscle [35,39,40,41]. These studies used different routes of cell administration with variable outcomes, mostly modest, with only one showing improvement of respiratory phenotype after systemic intraosseous transplantation of human chimeric myoblasts into mdx mice [40]. This scarcity of publications and limited engraftment are probably due to the difficulty of injecting cells directly into the diaphragm muscle.

In this study, we report the development of a reliable protocol to deliver muscle stem cells (also known as satellite cells) directly into the diaphragm. We applied this cell transplantation method to mdx and FKRP^P448L^ mice and showed rescue of the dystrophic phenotype in both models. In addition, force measurements in transplanted mdx mice showed that myofiber engraftment was accompanied by enhanced diaphragm muscle force.

## 2. Results

### 2.1. Optimization of a Method to Deliver Satellite Cells into the Diaphragm

**Figure 1:**
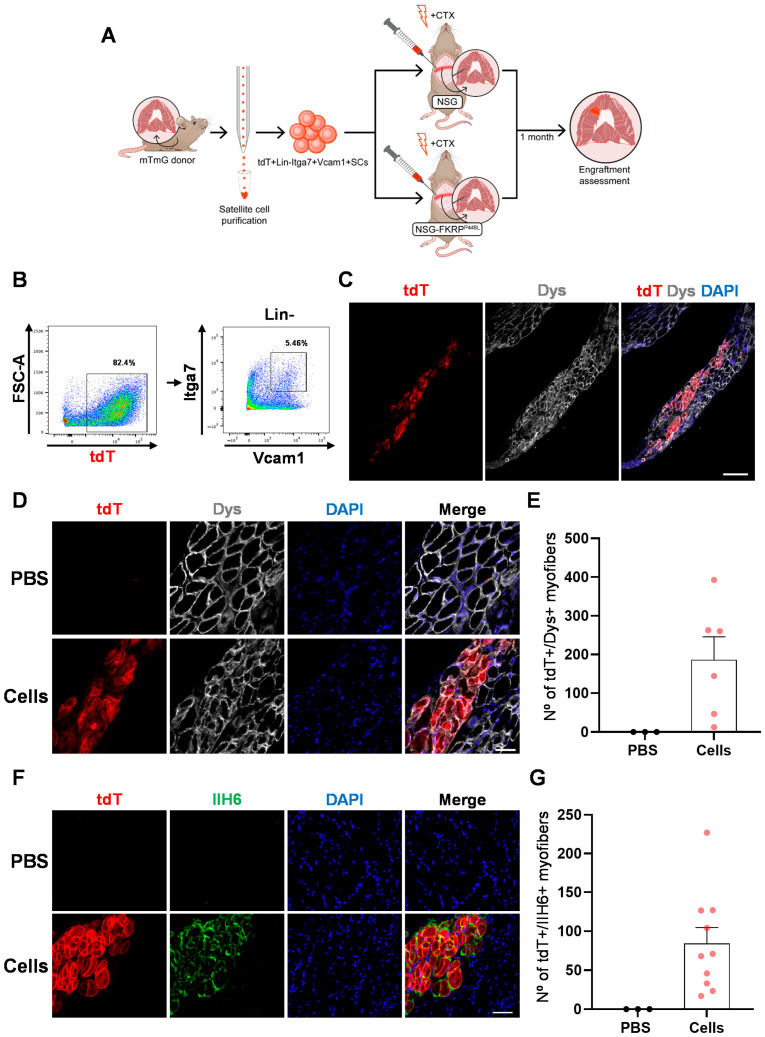
Detection of donor-derived myofibers in diaphragm muscles of NSG and NSG-FKRP^P448L^ mice. (**A**) Schematic representation outlining the isolation of satellite cells (SCs) from the diaphragm of a mTmG mouse and their transplantation into the diaphragm muscles of NSG and NSG-FKRP^P448L^ mice. (**B**) Representative FACS plots show SC isolation from mTmG mice based on the expression of Itga7 and Vcam1, following gating of the tdT+Lin- fraction. (**C**–**E**) Diaphragm transplantation in NSG mice. Representative images show the whole area of engraftment, as indicated by immunostaining for tdT (in red), dystrophin (Dys) (in gray), and DAPI (in blue). Scale bar is 200 µm (**C**). Representative images show the same staining at higher magnification for PBS- (upper panel) and SC-injected (lower panel) diaphragm muscles. Scale bar is 50 µm (**D**). Graph shows quantification of engraftment ((**E**) from (**D**)) based on the number of tdT+Dys+ myofibers. Data are shown as mean ± SEM (*n* = 3 for PBS and *n* = 6 for cells). (**F**,**G**) Diaphragm transplantation in NSG-FKRP^P448L^ mice. Representative images show staining for tdT and IIH6 in PBS- (upper panel) and SC-injected (lower panel) diaphragm muscles. Scale bar is 50 µm (**F**). Respective quantification of engraftment (**G**), as shown by the number of tdT+IIH6+ myofibers. Data are shown as mean ± SEM (*n* = 3 for PBS and *n* = 10 for cells).

To determine our ability to deliver cells directly into the diaphragm muscle, we chose to transplant satellite cells (SCs), since as bona fide muscle stem cells, these are endowed with exceptional regenerative capacity [42]. Previous studies reporting SC transfer into hind limb muscles demonstrated that engraftment is enhanced by muscle conditioning prior to transplantation [28,43]. Therefore, we adapted a protocol consisting of a combination of local muscle irradiation and cardiotoxin (CTX) injection [28]. We first validated this dual conditioning strategy in the diaphragm of wild-type (WT) immunodeficient NSG mice, as shown by clear signs of muscle damage at 7 and 30 days post-injury (Figure S1). For cell delivery, we implemented a laparotomy protocol, previously described in rats [44,45], that allows direct visualization of the diaphragm, enabling accurate intramuscular (IM) injections. To avoid performing two major surgeries, we injected CTX and SCs a few minutes apart within the same laparotomy procedure. SCs isolated from the diaphragms of mTmG transgenic mice, in which all cells express tdTomato (tdT) [46], were injected to facilitate tracking of donor-derived cells (Figure 1A). The SC fraction was isolated by fluorescence-activated cell sorting (FACS) based on the absence of CD31 and CD45, endothelial and hematopoietic markers, respectively (referred as lineage negative; Lin-), and the expression of the surface markers integrin α-7 (Itga7) and Vcam1 (Figure 1B), as previously reported [47]. Purified SCs were injected directly into the diaphragm of NSG mice. One month after transplantation, we detected the presence of donor-derived tdT+ myofibers expressing dystrophin (Dys) (Figure 1C,D). Quantification revealed an engraftment size of 186.5 ± 59 tdT+Dys+ myofibers (Figure 1E), which is significant considering the small injected area (approximately 5% of the diaphragm).

### 2.2. SC Transplantation into the Diaphragm Muscle of FKRP-Mutant Dystrophic Mice

Having confirmed the feasibility of this method to deliver cells directly into the diaphragm, we next applied this transplantation strategy (Figure 1A) to the NSG-FKRP^P448L^ mouse model, which combines the FKRP mutation with the immunodeficient background [48], thus avoiding the need for immunosuppression upon transplantation. For assessment of engraftment, we stained diaphragm cryosections with tdT and IIH6 antibodies. The IIH6 antibody specifically identifies functional glycosylation of α-dystroglycan (α-DG) [49,50], which is absent in FKRP-mutant mice [51]. Consistently, our results show the presence of myofibers double-positive for tdT and IIH6 in the transplanted diaphragm (Figure 1F,G), while PBS-injected counterparts were absent of IIH6 immunoreactivity (Figure 1F,G), confirming rescue of α-DG functional glycosylation. In addition to myofiber engraftment, we detected the presence of donor-derived SCs in the transplanted diaphragm, as indicated by the presence of tdT+Pax7+ cells under the basal lamina (Figure S2).

**Figure 2:**
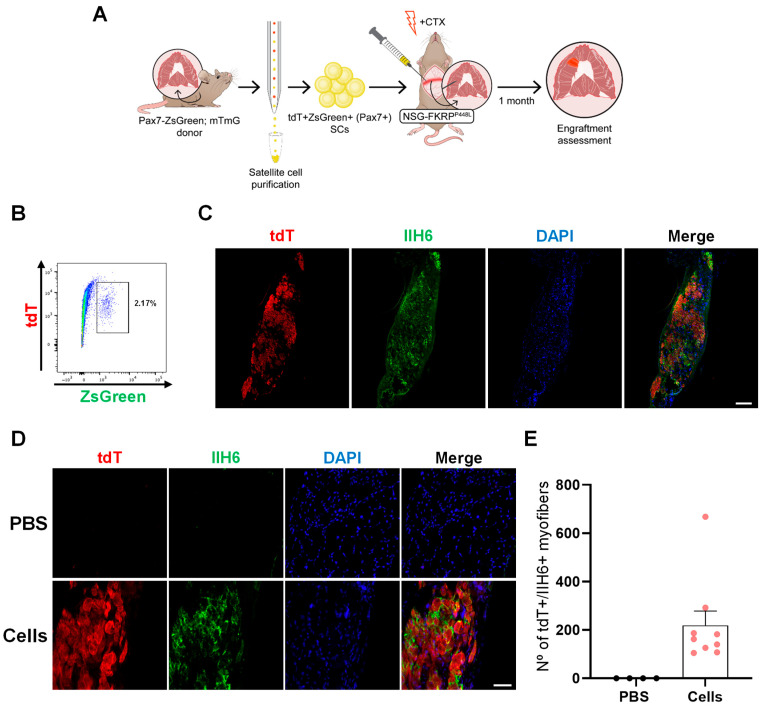
Engraftment of SCs isolated from Pax7-ZsGreen–mTmG mice in the diaphragm of NSG-FKRP^P448L^ mice. (**A**) Scheme outlines the isolation of SCs from Pax7-ZsGreen–mTmG mice and their transplantation into diaphragm muscles of NSG-FKRP^P448L^ mice. (**B**) Representative FACS plots show SC isolation from Pax7-ZsGreen–mTmG mice based on the expression of ZsGreen. (**C**,**D**) Representative images show diaphragm engraftment as indicated by immunostaining for tdT (in red), IIH6 (in green), and DAPI (in blue). Scale bar is 200 µm (**C**). (**D**) shows higher magnification of control (upper panel) and transplanted (lower panel) diaphragms. Scale bar is 50 µm. (**E**) Graph shows engraftment quantification (**D**), as shown by the number of tdT+IIH6+ myofibers. Data are shown as mean ± SEM (*n* = 4 for PBS and *n* = 9 for cells).

To expedite the purification process, we crossed Pax7-ZsGreen mice [52] with mTmG mice, so SCs could be isolated based on the expression of ZsGreen, which identifies Pax7+ SCs [52]. Using this purification approach, we proceeded with diaphragm transplantation into NSG-FKRP^P448L^ mice (Figure 2A,B). Immunofluorescence staining, one month after injection, confirmed the contribution of tdT+Pax7+ cells to muscle regeneration, as shown by the presence of tdT+IIH6+ myofibers (Figure 2C,D). Enumeration of donor-derived engraftment revealed 219 ± 59 myofibers (Figure 2E), representing 5 ± 1% of the whole diaphragm muscle (Figure S3A,B).

### 2.3. SC Transplantation Restores Dystrophin Expression in the DMD Mouse Diaphragm

**Figure 3:**
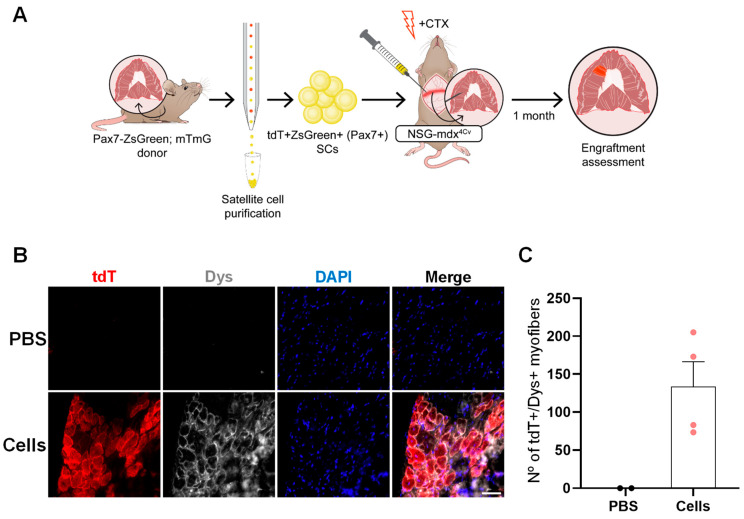
Rescue of dystrophin expression upon SC transplantation into the diaphragm of NSG-mdx^4Cv^ mice. (**A**) Schematic representation of transplantation outline. (**B**) Representative images show the presence of tdT+Dys+ (red and gray, respectively) myofibers in only the transplanted diaphragm muscles (lower panel). DAPI in blue-stained nuclei. Scale bar is 50 µm. (**C**) Graph shows respective engraftment quantification (**B**). Data are shown as mean ± SEM (*n* = 2 for PBS and *n* = 4 for cells).

Next, we sought to confirm the feasibility of our diaphragm transplantation approach by injecting SCs into the NSG-mdx^4Cv^ mouse model of DMD (Figure 3A), which is commonly used for cell transplantation studies [27,28,47,53,54]. As shown in Figure 3B,C, the delivery of tdT+ZsGreen+ SCs into the diaphragm of NSG-mdx^4Cv^ resulted in rescue of dystrophin expression, as indicated by the presence of tdT+Dys+ donor-derived myofibers (134 ± 33), corresponding to approximately 5 ± 1% of the whole diaphragm (Figure S4A,B).

### 2.4. Diaphragm Targeted Cell Therapy Improves Muscle Force

**Figure 4:**
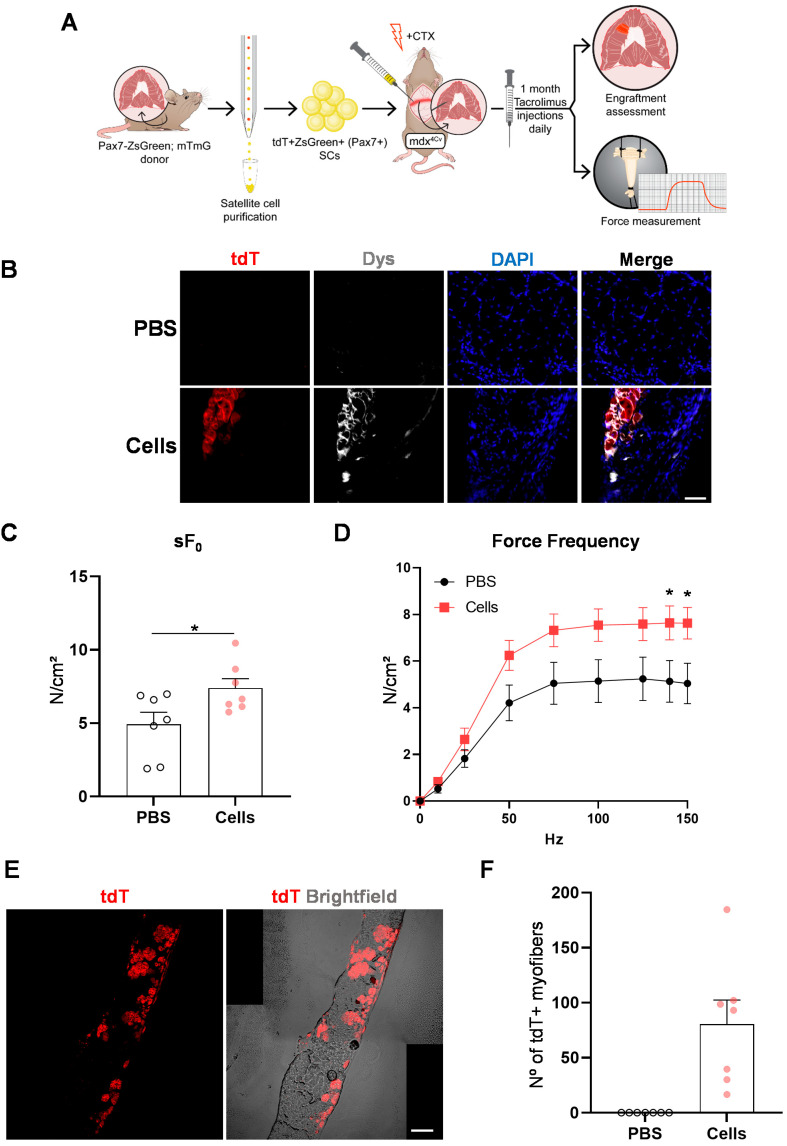
Diaphragm engraftment results in improvement of muscle force in mdx^4Cv^ mice. (**A**) Scheme indicates the outline of transplantation into the diaphragm muscles of immunocompetent mdx^4Cv^ mice undergoing tacrolimus immunosuppression. One month later, the diaphragm was assessed for engraftment and force measurement. (**B**) Representative images show immunostaining for tdT (in red), Dys (in gray), and DAPI (blue) for PBS-injected (top panel) and cell-injected (lower panel) diaphragms. Scale bar is 50 µm. (**C**) Specific (sF_0_: maximum force normalized to CSA) force of cell-injected diaphragm compared with PBS. Data are shown as mean ± SEM (*n* = 7 for PBS and cells). * *p* < 0.05 according to *t* test. (**D**) Specific force measured at different stimulation frequencies, comparing cells with PBS. Data are shown as mean ± SEM (*n* = 7 for each group). * *p* < 0.05 according to *t* test. (**E**) Representative images show engraftment in a cell-injected diaphragm strip, as indicated by the presence of tdT (in red). Brightfield is indicated in gray. Scale bar is 100 µm. (**F**) Graph shows quantification of the number of tdT+ myofibers present in transplanted diaphragm strips. Data are shown as mean ± SEM (*n* = 7 for each group).

To mimic clinical translation, we transplanted tdT+ZsGreen+ SCs into the diaphragm muscles of immunocompetent mdx^4Cv^ mice undergoing immunosuppression with tacrolimus (Figure 4A). Again, we observed rescue of dystrophin expression, as evidenced by the presence of tdT+Dys+ myofibers one month after transplantation (Figure 4B). To determine whether engraftment resulted in functional improvement, we performed ex vivo force measurements of diaphragm strips [55] that had been injected with SCs or PBS. As shown in Figure 4C, the specific force was superior in cell-injected strips compared with PBS. We observed the same trend when diaphragm strips were submitted to a series of tetanic stimulation at increasing frequencies (Figure 4D). Importantly, we confirmed the presence of tdT+ myofibers (81 ± 22) in the diaphragm strips used for force measurements (Figure 4E,F), therefore corroborating that diaphragm engraftment with SCs led to improvement in muscle force in mdx mice.

## 3. Discussion

There have been only a few studies reporting diaphragm muscle targeting with cell transplantation. This is probably due to the complexity of reaching and injecting cells into this muscle. Targeting the diaphragm with IM injections is not a trivial procedure, and thus other administration routes have been explored. In 2013, Filareto and colleagues detected the presence of donor-derived myofibers in the diaphragm following the intravenous transplantation of murine pluripotent stem cell-derived myogenic progenitors in mdx/utrophin^−/−^ mice [35]. A recent report showed that the systemic intraosseous delivery of human chimeric cells obtained by the fusion of bone marrow cells and myoblasts in mdx mice could effectively target the diaphragm, as 15% dystrophin expression was detected, which was accompanied by improvement in respiratory capacity [40]. In terms of direct injection, one publication documented reaching the diaphragm through laparoscopy, which allowed access to this muscle using small abdominal incisions, but it required a complex set up. This approach was used to deliver mesenchymal stem cells into the diaphragm muscle of mdx mice. Even though transplanted cells were detectable eight days after injection, there was no myofiber contribution nor any clear benefit since these cells are not endowed with myogenic potential [41]. Laparotomies have been more commonly used, which consist of a large abdominal incision to allow IM delivery. Different injection methods have been documented [44,45,56,57,58]. Here, we injected SCs perpendicularly into the diaphragm, as previously described for the retrograde labeling of phrenic motoneurons to successfully deliver tetramethylrhodamine-conjugated dextran into the diaphragm [44,45]. For the scope of the present study, we restricted the injections to a ~4 mm midcostal diaphragm sector, but one can envision injecting a larger area for translational purposes.

A recent publication used a laparotomy approach to deliver human induced pluripotent stem cell-derived myogenic progenitors to the edge of the mouse diaphragm muscle. Nonetheless, human contribution was very limited (fewer than two engrafted fibers), and this outcome was just slightly improved when cells were co-injected with a polymer, with the intention to retain the cells at the injection site. To validate their delivery method, the authors also transplanted SCs, which resulted in an engraftment of approximately 40 donor-derived fibers, but neither functional improvement nor the presence of donor-derived SCs were reported [39].

Here, we provide a method for muscle stem cell transplantation into the diaphragm with a direct impact on muscle force. We have shown that the pre-injury model consisting of irradiation combined with CTX, widely used for pre-conditioning of hind limbs [28,47,59,60], can be applied to the diaphragm. The generation of Pax7-ZsGreen–mTmG mice facilitated the process for SC isolation to engraftment detection. We showed that SC diaphragm transplantation produces donor-derived myofibers that rescue the phenotype of both LGMD2I and DMD mouse models, as shown by the presence of IIH6+ and Dys+ myofibers, respectively. We also detected the presence of donor-derived SCs. Importantly, engraftment improved diaphragm contractility.

Future directions to confirm the therapeutic potential of this approach would in-clude (i) the assessment of long-term engraftment to ensure persistent benefits, as ob-served in limb muscles [48,61,62,63,64], (ii) the testing of different cell types, such as pluripotent stem cell-derived myogenic progenitors, which possess no limitations on cell numbers [26,27], (iii) transplantation in older dystrophic mice, in which the diaphragm is more affected [65,66], and importantly, (iv) the injection of larger areas to produce more robust therapeutic benefit, which would increase the likelihood of improvement of respiratory function in dystrophic recipients. 

## 4. Materials and Methods

### 4.1. Mice

All animal studies were performed according to protocols approved by the University of Minnesota Institutional Animal Care and Use Committee. Pax7-ZsGreen [52] and mTmG mice [46] were crossed to generate Pax7-ZsGreen–mTmG mice. SCs were purified from 4-to-6-week-old mTmG and Pax7-ZsGreen–mTmG mice. For cell transplantation, we utilized the following mouse strains at six to eight weeks of age: NSG (Jackson Laboratories, BarHarbor, ME, USA), NSG-FKRP^P448L^ [48], NSG-mdx^4Cv^ [28], and mdx^4Cv^ (Jackson Laboratories). For NSG, NSG-mdx^4Cv^, and mdx^4Cv^ mice, only males were used. For the NSG-FKRP^P448L^ mouse strain, both males and females were used.

### 4.2. SC Isolation

Diaphragm muscles collected from mTmG or Pax7-ZsGreen–mTmG mice were chopped, resuspended in high-glucose DMEM (Gibco, Waltham, MA, USA) containing 2 mg/mL collagenase II (Gibco) and 1% penicillin/streptomycin (Invitrogen, Waltham, MA, USA), and digested for an hour in a 37 °C shaker. Then, one volume of PBS was added and the digested tissue was centrifuged at 500× *g* for 10 min. The pellet was washed twice with rinsing solution, which consisted of Ham’s/F-10 media (Hyclone, Logan, UT, USA) containing 10% horse serum (Hyclone), 1% penicillin/streptomycin (Invitrogen), and 0.1 μM HEPES (Gibco), at 500× *g* for 7 min. The remaining tissue was shredded with a Pasteur pipette and submitted to a second round of digestion with 0.1 mg/mL collagenase II (Gibco) and 0.5 mg/mL dispase (Gibco) in rinsing solution for 30 min at 37 °C. The solution was then homogenized with 16 and 18 G needles, filtered with a 40 µm cell strainer, and spun down at 700× *g* for 7 min. Mononuclear cells (MNCs) were resuspended in FACS buffer, which consisted of PBS (Gibco) containing 10% FBS (Sigma, Burlington, MA, USA) and 1% penicillin/streptomycin (Invitrogen), and used for SC isolation. SCs from Pax7-ZsGreen–mTmG mice were sorted based on ZsGreen (Pax7) expression, whereas SCs from mTmG mice were purified based on the absence of lineage markers (Lin-) CD31 (endothelial) and CD45 (hematopoietic) and expression of the surface markers Itga7 and Vcam1 [47]. MNCs were stained with anti-CD31 and anti-CD45 (both PE-Cy7-conjugated, from eBiosciences), anti-Itga7 (APC-conjugated, AbLab, Vancouver, BC, Canada), and anti-biotin-Vcam1 (eBiosciences, San Diego, CA, USA) antibodies for 20 min on ice. After incubation, cells were centrifuged at 1000× *g* for 5 min, stained with the PE-Cy7-conjugated streptavidin secondary antibody (eBiosciences) for 10 min on ice, and centrifuged again at 1000× *g* for 5 min. After one more PBS wash, cells were resuspended in FACS buffer and sorted with the FACS Aria II.

### 4.3. Diaphragm Cell Transplantation

One day prior to transplantation, recipient mice were anesthetized with ketamine/xylazine at 80 mg/kg by intraperitoneal (IP) injection and their diaphragms were irradiated with 10 Gy [67]. For diaphragm injections, the same ketamine/xylazine combination was used to anesthetize the mice. As previously described [44,45], a laparotomy was performed to expose the diaphragm. We injected 30 µL of 10 µM CTX (Latoxan, Porte les Valences, France) followed by 5000 FACS-purified SCs resuspended in 30 µL of PBS. Diaphragms injected with PBS alone served as a negative control. Five to eight IM injections were performed in the top right part of the diaphragm muscle using a 33 g Hamilton syringe. For immunosuppression, a dose of 5 mg/kg of the immunosuppressive agent tacrolimus (MedChemExpress, Monmouth Junction, NJ, USA) was injected daily via IP to the immunocompetent mdx^4Cv^ recipients. Treatment started one day before irradiation and ended by the day of euthanasia [26]. Diaphragm muscles were collected for engraftment assessment at 4 weeks post-transplantation.

### 4.4. Immunofluorescence and H&E Staining

Tissue-Tek O.C.T. compound (Sakura, Torrance, CA, USA) was used to embed dissected diaphragm muscles. These were then snapped frozen on isopentane pre-cooled with liquid nitrogen, and 14 µm cryosections were cut and kept at −80 °C. Before staining, muscle samples were rehydrated with PBS for 5 min at room temperature (RT). The fixation step consisted of 4% PFA for 30 min at RT, and cryosections were then washed with PBS. For the permeabilization, 0.3% Triton X100 (Sigma) in PBS was applied for 15 min at RT. Samples were then washed again with PBS, blocked for 30 min with 3% BSA (Sigma), and incubated overnight at 4 °C with primary antibodies. Primary antibodies included RFP (rabbit 1:500, ab62341 Abcam, Cambridge, UK), Dys (mouse 1:20, DYS1-CE Leica and rabbit 1:200, ab15277 Abcam), laminin α-2 (Lam, rat 1:200, Sc-59854 Santa Cruz, Dallas, TX, USA), Pax7 (mouse 1:10, DSHB), and IIH6 (mouse 1:200, 05-593 Millipore, Burlington, MA, USA). Next, muscle cryosections were rinsed with PBS, and stained with Alexa Fluor 555 anti-rabbit immunoglobulin G (IgG) (A-21428; 1:500), Alexa Fluor 647 anti-mouse IgG (A-21236; 1:500), Alexa Fluor 647 anti-rabbit IgG (A-21245; 1:500), Alexa Fluor 488 anti-rat IgG (A-11006; 1:500) (all from Thermo Fisher Scientific, Waltham, MA, USA) and 4,6-Diamidino-2-phenylindole (DAPI, Santa Cruz) for 1 hr at RT. Cryosections were then washed 3 times with PBS and mounted with Prolong Gold (Invitrogen). H&E staining was performed using the SelecTech kit (Leica Biosystems, Wetzlar, Germany). Slides were analyzed with upright (Zeiss, Oberkochen, Germany) and confocal microscopy (NikonNiE C2, Nikon Instruments Inc., Melville, NY, USA). Image processing and quantification were performed with Fiji software v1.54f.

### 4.5. Force Measurements

For the measurements of diaphragm contractile force, we adapted a previously described protocol [55]. We dissected ~4 mm midcostal diaphragm strips and placed these in the organ bath of the 3-in-1 animal system (Aurora Scientific Inc., Aurora, ON, Canada) filled with mammalian Ringer solution containing 120.5 mM NaCl, 20.4 mM NaHCO_3_, 10 mM dextrose, 4.8 mM KCl, 1.6 mM CaCl_2_, 1.2 mM MgSO_4_, 1.2 mM NaH_2_PO_4_, and 1.0 mM pyruvate. Upon dissection and force measurement, the solution was perfused continuously with 95% O_2_–5% CO_2_ and maintained at 25 °C. Diaphragm strips were then set to optimal length, maximum force (F_0_) was meassured by stimulating for 1000 ms at 120 Hz. Specific force (sFo) was calculated by normalizing maximum force with the division of the diaphragm strip mass (mg) by the product of diaphragm strip length (mm) and 1.06 mg/mm^3^ as the density of skeletal muscle. The force frequency was determined by stimulating the diaphragm strip with 1000 ms trains of 0.5 ms stimuli at 10, 25, 50, 75, 100, 125, 140, and 150 Hz.

### 4.6. Statistical Analysis

Differences between the transplanted and PBS groups were assessed using the *t*-test for independent samples; *p*-values lower than 0.05 were considered significant. Statistical analyses were performed using GraphPadPrism v9.1.2 (GraphPadSoftware, LLC, San Diego, CA, USA).

## Data Availability

The data supporting this article are found within the text and the supplementary information file. Any additional data and the data that support the plots within this paper are available from the corresponding author upon reasonable request.

## Supplementary Materials

The following supporting information can be downloaded at: https://www.mdpi.com/article/10.3390/ijms25052503/s1.
