# Supplementary material for: Enhanced Diaphragm Muscle Function upon Satellite Cell Transplantation in Dystrophic Mice
Source: Int J Mol Sci. 2024 Feb 21;25(5):2503. doi: 10.3390/ijms25052503 (PMC10931593; doi:10.3390/ijms25052503)
Supplement: Supplementary file 1 [file ijms-25-02503-s001.zip › Supplementary Figures 011324.pptx]

## Slide 1
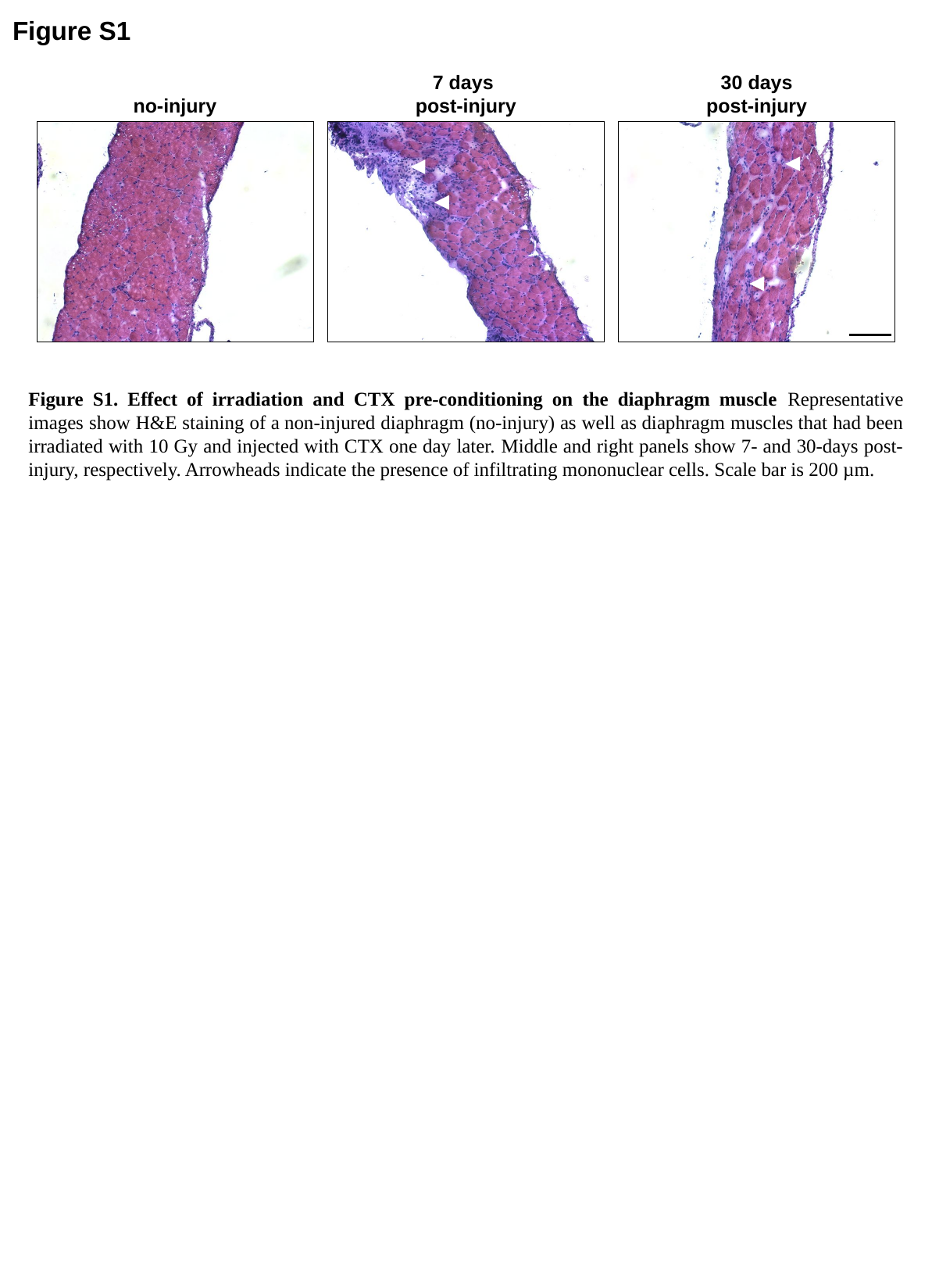

Figure S1
7 days
post-injury
30 days
post-injury
no-injury
Figure S1. Effect of irradiation and CTX pre-conditioning on the diaphragm muscle Representative images show H&E staining of a non-injured diaphragm (no-injury) as well as diaphragm muscles that had been irradiated with 10 Gy and injected with CTX one day later. Middle and right panels show 7- and 30-days post-injury, respectively. Arrowheads indicate the presence of infiltrating mononuclear cells. Scale bar is 200 µm.

## Slide 2
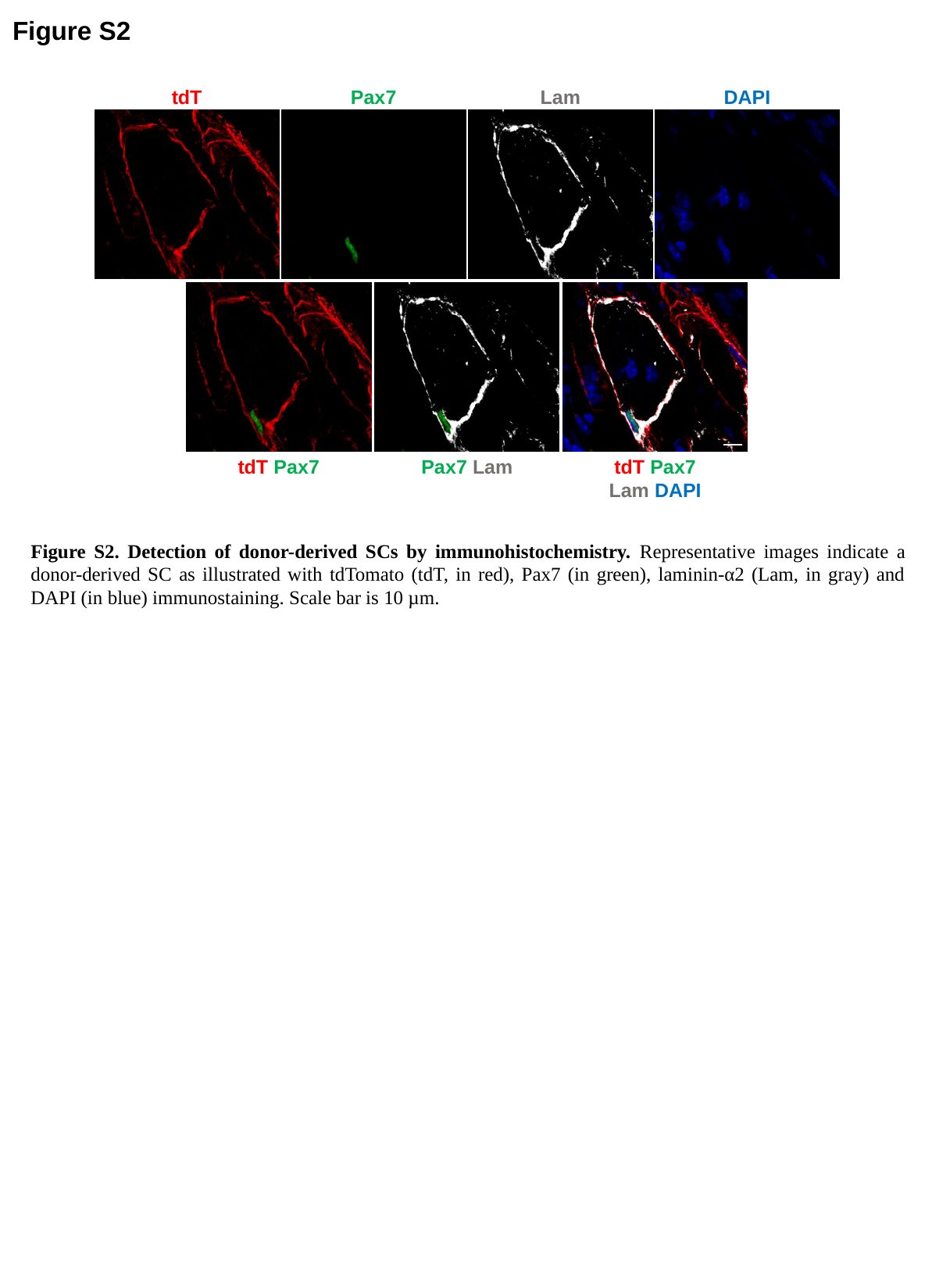

Figure S2
tdT
Pax7
Lam
DAPI
tdT Pax7
Pax7 Lam
tdT Pax7
Lam DAPI
Figure S2. Detection of donor-derived SCs by immunohistochemistry. Representative images indicate a donor-derived SC as illustrated with tdTomato (tdT, in red), Pax7 (in green), laminin-α2 (Lam, in gray) and DAPI (in blue) immunostaining. Scale bar is 10 µm.

## Slide 3
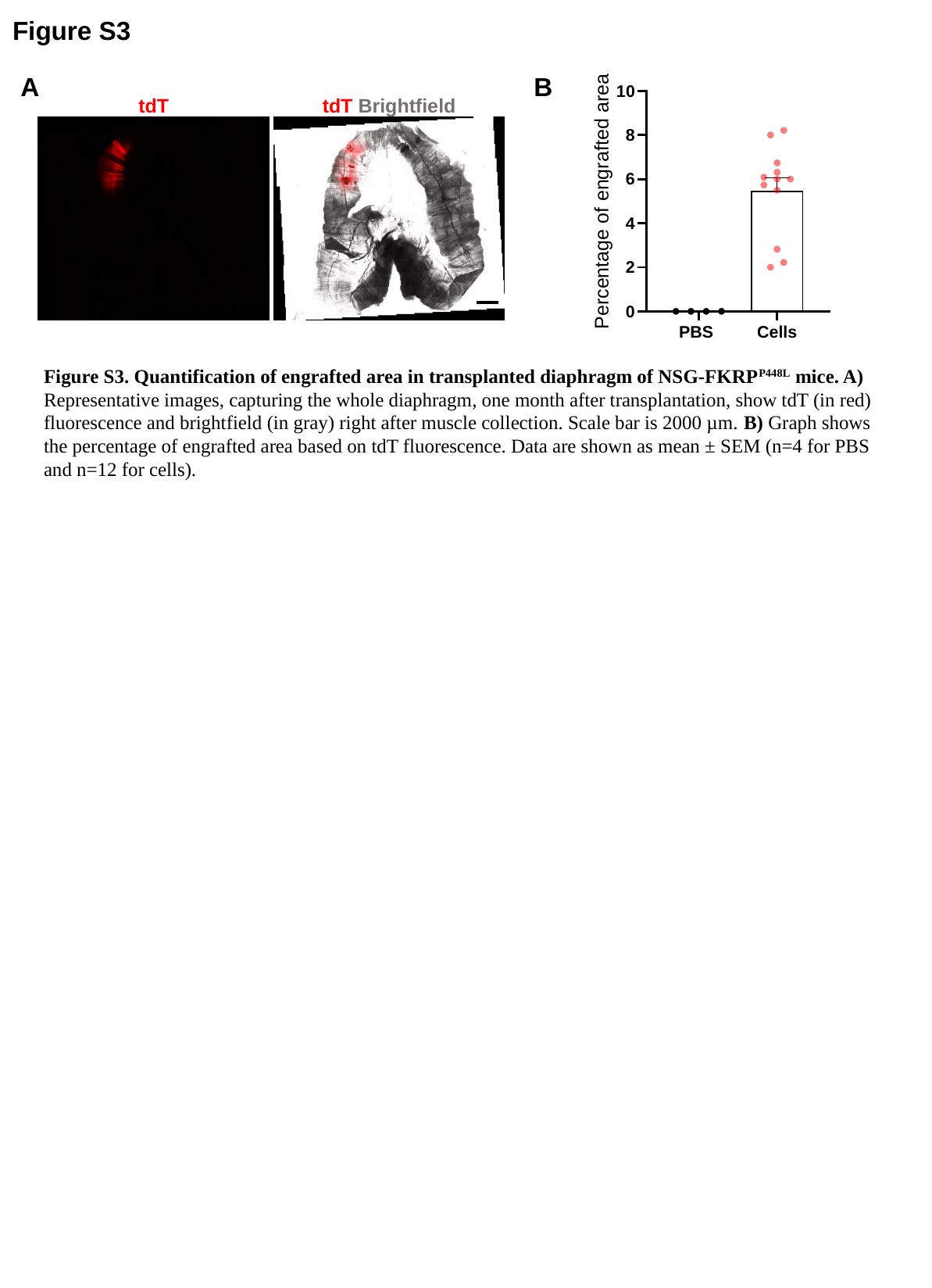

Figure S3
A
B
tdT
tdT Brightfield
Percentage of engrafted area
Figure S3. Quantification of engrafted area in transplanted diaphragm of NSG-FKRPP448L mice. A) Representative images, capturing the whole diaphragm, one month after transplantation, show tdT (in red) fluorescence and brightfield (in gray) right after muscle collection. Scale bar is 2000 µm. B) Graph shows the percentage of engrafted area based on tdT fluorescence. Data are shown as mean ± SEM (n=4 for PBS and n=12 for cells).

## Slide 4
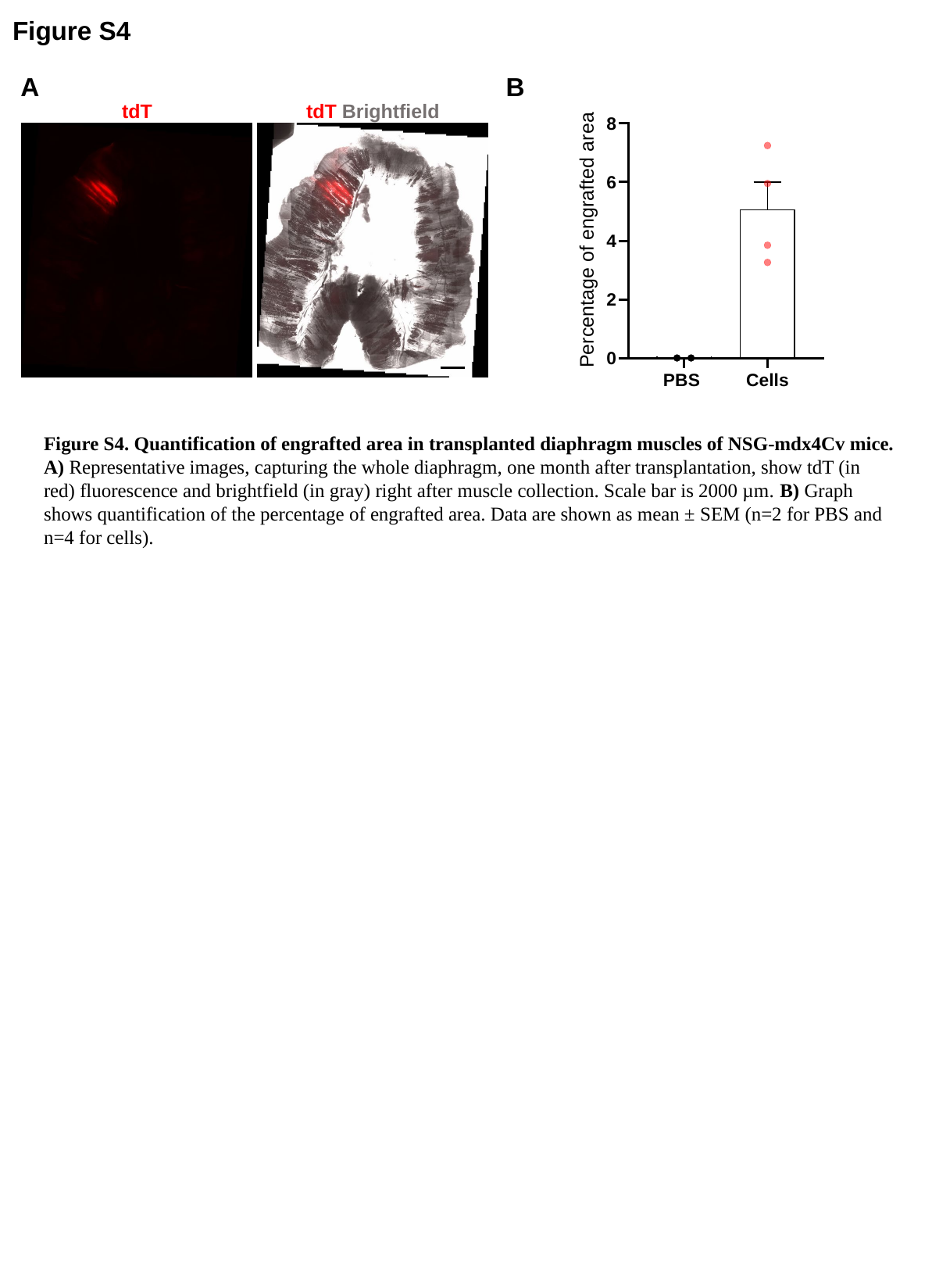

Figure S4
A
B
tdT
tdT Brightfield
Percentage of engrafted area
Figure S4. Quantification of engrafted area in transplanted diaphragm muscles of NSG-mdx4Cv mice. A) Representative images, capturing the whole diaphragm, one month after transplantation, show tdT (in red) fluorescence and brightfield (in gray) right after muscle collection. Scale bar is 2000 µm. B) Graph shows quantification of the percentage of engrafted area. Data are shown as mean ± SEM (n=2 for PBS and n=4 for cells).
